# Bacterial Outer Membrane Vesicles (OMVs)-Based Dual Vaccine for Influenza A H1N1 Virus and MERS-CoV

**DOI:** 10.3390/vaccines7020046

**Published:** 2019-05-28

**Authors:** Mahmoud M. Shehata, Ahmed Mostafa, Lisa Teubner, Sara H. Mahmoud, Ahmed Kandeil, Rabeh Elshesheny, Thamer A. Boubak, Renate Frantz, Luigi La Pietra, Stephan Pleschka, Ahmed Osman, Ghazi Kayali, Trinad Chakraborty, Mohamed A. Ali, Mobarak Abu Mraheil

**Affiliations:** 1 Center of Scientific Excellence for Influenza Viruses, Environmental Research Division, National Research Centre (NRC), Cairo 12622, Egypt; Mahmoud.Shehata@human-link.org (M.M.S.); ahmed_elsayed@daad-alumni.de (A.M.); sara.Hussein@human-link.org (S.H.M.); ahmed.Kandeil@human-link.org (A.K.); rabeh.elshesheny@stjude.org (R.E.); 2 Institute of Medical Virology, Justus-Liebig University Giessen, 35392 Giessen, Germany; Stephan.Pleschka@viro.med.uni-giessen.de; 3 Institute of Medical Microbiology, German Center for Infection Research (DZIF), Partner Site Giessen-Marburg-Langen Site, Justus-Liebig University Giessen, 35392 Giessen, Germany; lisa.teubner@mikrobio.med.uni-giessen.de (L.T.); renate.frantz@mikrobio.med.uni-giessen.de (R.F.); luigi.La-pietra@mikrobio.med.uni-giessen.de (L.L.P.); trinad.chakraborty@mikrobio.med.uni-giessen.de (T.C.); 4 Biological Department, Faculty of Science, King Abdul Aziz University, Jeddah 80203, Saudi Arabia; Th_ah@hotmail.com; 5 Department of Biochemistry, Faculty of Science, Ain Shams University, Cairo 38105, Egypt; aoegiza@yahoo.com; 6 Department of Epidemiology, Human Genetics, and Environmental Sciences, University of Texas, Houston, TX 77030, USA; ghazi@human-link.org; 7 Human Link, Baabda 1109, Lebanon

**Keywords:** OMVs, influenza vaccine, MERS-CoV, H1N1pdm

## Abstract

Vaccination is the most functional medical intervention to prophylactically control severe diseases caused by human-to-human or animal-to-human transmissible viral pathogens. Annually, seasonal influenza epidemics attack human populations leading to 290–650 thousand deaths/year worldwide. Recently, a novel Middle East Respiratory Syndrome Coronavirus emerged. Together, those two viruses present a significant public health burden in areas where they circulate. Herein, we generated a bacterial outer membrane vesicles (OMVs)-based vaccine presenting the antigenic stable chimeric fusion protein of the H1-type haemagglutinin (HA) of the pandemic influenza A virus (H1N1) strain from 2009 (H1N1pdm09) and the receptor binding domain (RBD) of the Middle East Respiratory Syndrome Coronavirus (MERS-CoV) (OMVs-H1/RBD). Our results showed that the chimeric antigen could induce specific neutralizing antibodies against both strains leading to protection of immunized mice against H1N1pdm09 and efficient neutralization of MERS-CoV. This study demonstrate that OMVs-based vaccines presenting viral antigens provide a safe and reliable approach to protect against two different viral infections.

## 1. Introduction

Acute respiratory infections are among the leading causes of disease and mortality in developing and developed countries [1,2]. The severity of these acute infections is usually potentiated following the dissemination of the infection throughout the lower respiratory tract, leading to millions of human deaths worldwide each year [3].

Annually, seasonal influenza epidemics attack 10–20% of the human population leading to 290–650 thousand deaths/year worldwide [4]. Beside these epidemics, the world is confronted every 10–40 years with antigenically distinct pandemic influenza virus strains of wide geographical distribution and considerable human-to-human transmissibility resulting in high mortality rates [5]. In recent years, the world has been challenged with newly emerging influenza A virus (IAV) infections, which have the potential to cause sporadic fatalities in the human population within limited epidemics. For instance, highly pathogenic avian influenza viruses (HPAIV) of the H5N1-subtype and low pathogenic avian influenza viruses (LPAIV) of the H7N9-, H10N8- and H6N1-subtypes [6,7,8,9] have caused sporadic human infections. In addition, other IAVs caused global pandemic outbreaks, such as the 2009 swine-origin H1N1 influenza virus (H1N1pdm09) [10,11].

In 2012, a novel Middle East Respiratory Syndrome Coronavirus (MERS-CoV) emerged. By February 2019, a total of 2374 laboratory-confirmed human cases, including 823 associated deaths, were reported globally in 27 countries (case-fatality rate: 35.4%) [12]. The majority of these cases were reported from the Arabian Peninsula, specifically Saudi Arabia (cases = 1896; deaths = 732; case-fatality rate = 38.6%) [12].

To combat IAV and MERS-CoV infections, vaccination represents an affordable and a facile way to protect against devastating epidemics and occasional pandemics. However, despite significant efforts to develop a safe and effective vaccine [13], there are no approved vaccines for MERS-CoV till now. Recent reports have also demonstrated that replication of recombinant IAV vaccine strains in either embryonated eggs or in cell-culture systems allows viral adaptation, which may affect the antigenicity of the vaccine [14,15,16]. Therefore, genetically and phenotypically stable vaccines represent a promising alternative to control IAV and MERS-CoV infections [14].

Outer membrane vesicles (OMVs) are natural, spherical nanoparticles (50–250 nm) derived from Gram-negative bacteria. OMVs are released from both pathogenic and non-pathogenic bacteria and are highly immunogenic due to their components, including lipopolysaccharides (LPS), bacterial outer membrane (OM) proteins, lipids, immunogenic toxins, DNA/RNA and other periplasmatic and cytoplasmatic proteins [17,18].

OMVs from pathogenic bacteria have been commercially used to induce specific antibodies against different bacterial strains, including *Neisseria meningitidis* serogroup B [19]. The composition of the OMVs can be adapted and used as a vaccine platform via incorporation of heterologous antigens into the vesicles [20]. This engineering approach is advantageous because (i) it retains the antigens in their native conformation, (ii) it enables the OMVs to target specific immune responses, and (iii) it provides multiple and commensurate protein antigens in a single production process [17]. However, bacteria-based vaccines are not well explored to deliver viral antigens. Therefore, we engineered a stable OMVs-based dual vaccine against H1N1pdm09 and MERS-CoV by producing OMVs with a chimeric hemagglutinin (HA) comprising of both HA1 and HA2 from the H1N1pdm09 and the receptor binding domain (RBD) of MERS-CoV.

## 2. Materials and Methods

### 2.1. Viruses and Plasmids

Influenza virus A/California/04/2009 (Cal-H1N1pdm2009) was kindly provided by Dr. Richard Webby, St Jude Children’s Research Hospital, Memphis, TN, USA. An Egyptian MERS-CoV isolate, MERS-CoV/Camel/Egypt/HKU-NRCE205/2013 (accession no. KJ477102), was obtained from the virus collection of Center of Scientific excellence for Influenza Viruses, National Research Centre, Egypt. The Cal-H1N1pdm2009 strain was propagated in Madin-Darby Canine Kidney (MDCK) cells, while the MERS-CoV strain was isolated and grown in Vero-E6 cells. The two viruses were used for preparation of the OMVs-based dual vaccine and inactivated vaccines were used as positive control.

### 2.2. Construction of Plasmids

To construct the pMP-H1/RBD plasmid, three PCR fragments (F1, F2, and F3) encompassing (i) 5’-NCR and signal peptide of HA from Cal-H1N1pdm2009, (ii) RBD of MERS-CoV and a 7-amino acid/peptide linker (GSAGSAG), (iii) the coding sequence and 3’-NCR of HA were amplified with sequence-specific primers (Table 1) and Phusion High-Fidelity PCR Master Mix with HF Buffer (Invitrogen, Carlsbad, CA) and then simultaneously ligated into linearized pMPccdB vector [21]. Briefly, for the PCR amplification of each fragment, 25 µL of 2× Phusion Master Mix, 2.5 µL of forward and reverse primers (10 µM/µL), and 50 ng of the according template DNA were mixed and the reaction was then brought to a total volume of 50 µL using RNase-/DNase-free ddH_2_O. The plasmid pMP-HA-Gi [21] encoding the HA of Cal-H1N1pdm2009 was used as template DNA for F1 and F3, while the plasmid pcDNA3.1-Spike-MERS-CoV encoding the spike protein from the isolate MERS-CoV/Camel/Egypt/HKU-NRCE-270/2013 was used as a template for F2. The PCR settings were: 95 °C for 1 min then 3 steps of 40 cycles (95 °C for 10 s, 56 °C for 30 s, and 72 °C for 2 min), with a final extension step at 72 °C for 10 min. The three amplified PCR fragments were then loaded onto a 1% agarose-gel for electrophoresis. Separation and purification of the three specific fragments was done by using QIAquick gel purification kit according to manufacturer`s (Qiagen, Germany) instructions. After purification the three fragments were digested by corresponding restriction enzymes, shown in Table 1.

Ligation of the three fragments and the linearized vector was performed using T4 DNA ligase (Promega, Madison, WI, USA) by adding 5 µL of each purified fragment (20 ng/µL) to 2 µL 10× buffer, 2 µL T4 DNA ligase, and 1 µL linearized vector (20 ng/µL). The mixture was then incubated overnight at 4 °C. Transformation of *Escherichia* coli DH5-α competent cells was performed by mixing 5 µL of the ligation reaction with 50 µL bacterial suspension (Invitrogen, CA, USA) and subsequent incubation on ice for 30 min. The bacterial cells were then subjected to heat shock at 42 °C for 30 s in a water bath and were then chilled on ice for 2 min before adding 250 µL of SOC media (Invitrogen, CA, USA). The reaction tubes were then rotated at 250 rpm in a shaking incubator at 37 °C for 1 h. After an incubation time of 1 h, 100 µL of the transformed bacterial suspension was spread on ampicillin containing Luria-Bertani (LB) Agar plate and incubated for 16 h at 37 °C. Single colonies were then selected and incubated in 5 mL liquid LB for 16 h for subsequent plasmid isolation and the correct sequence was verified by sequencing using a Big Dye Terminator Kit 3.1 (Thermofisher, CA, USA) at Macrogen facility (Seoul, South Korea).

### 2.3. Purification and Quantification of OMVs

*E. coli* DH10ß competent bacteria (Invitrogen, CA, USA) were transformed with pMP-H1/RBD according to manufacturer’s instructions as described above with 20 ng of pMP-H1/RBD plasmid. Individual colonies from ampicillin containing LB Agar plate were picked and incubated in 5 mL liquid LB for 16 h. These cultures were then used to inoculate the large-volume cultures in the next step.

OMVs are typically purified from supernatants of transformed *E. coli* DH10ß cells. To this point, 2 liters (4 × 500 mL) of DH10ß cultures (inoculated with 5 mL of the starter culture) were grown in LB broth at 37 °C in an orbital shaking incubator at 180 rpm until reaching the exponential phase (OD_600nm_ 1.0). The grown bacteria were pelleted at 6000× *g* for 15 min and the supernatant was sterile-filtered (Millipore Express PLUS Membrane Filter, PES, 0.22 µm) to remove residual bacteria. Afterwards, the bacteria free supernatant was concentrated by ultrafiltration using KrosFlo Research II TFF and a 100 kDa hollow fiber membrane (Spectrum Labs, Germany) to a final volume of 30 mL. The resulting filtrate (30 mL) was subjected to further ultracentrifugation at 150,000× *g* for 3 h and 4 °C in a SW41 Ti rotor (Beckman, GA, USA) to separate the OMVs fraction. Subsequently, the OMVs containing pellet was resuspended in 300 µL PBS (Dulbecco’s Phosphate Buffered Saline, Biochrom GmbH), sterile filtered (Millex-GV Syringe Filter Unit, PVDF, 0.22 µm) and stored at −80 °C until use. The amount of isolated OMVs was quantified by protein concentration measurement using Bradford protein assay.

### 2.4. Immunoblotting Analysis

Enriched OMVs (5 µg) from cultured DH10ß, either transformed with empty vector (control) or with pMP-H1/RBD, were mixed with 10 µL 4×  SDS sample buffer (40% glycerol, 240 mM Tris/HCl (pH 6.8), 8% SDS, 0.04% bromophenol blue, and 5% β-mercaptoethanol) and incubated for 5 min at 95 °C. The OMVs samples were then separated on precast gradient NuPAGE^®^ Novex^®^ 4–12% Bis-Tris protein gels (Invitrogen, USA) and subsequently transferred onto immobilon-FL polyvinylidene fluoride (PVDF) membranes (Merck Millipore). Following protein transfer, the PVDF membrane was blocked using blocking buffer (1× TBS (20 mM Tris-HCl, pH 7.6, 140 mM NaCl) containing 5% non-fat dry milk) for 1 h at room temperature (RT). The membrane was washed once for 5 min using washing buffer (1× TBS-Tween (20 mM Tris-HCl, pH 7.6, 140 mM NaCl, 0.05% Tween20)). Afterwards, detection of the viral HA1 protein was achieved using rabbit monoclonal antibodies recognizing influenza A virus hemagglutinin (Abcam), diluted 1:1000 in blocking buffer. 1 h later, the membrane was washed three times for 5 min with washing buffer. Next, the membranes were incubated in the dark for 1 h with the corresponding goat anti-rabbit IRDye (LI-COR, Nebraska, USA), diluted 1:10,000 in blocking buffer containing a 1:1000 dilution of 10% SDS. After three washing steps (5 min each), twice with washing buffer and once with 1× TBS, the proteins were visualized using an Odyssey Infrared Imaging System and application software package (LI-COR, Nebraska, USA).

### 2.5. Preparation of Inactivated Control Vaccines

The propagated virus was inactivated using 0.1% formaldehyde in 4 °C for 24 h. To ensure that there are no active viral particles following inactivation process of the inactivated control vaccines, MDCK and Vero E6 cells were inoculated with 100 µL of the inactivated strains. About 72 h post-inoculation, the cell-culture supernatant was then tested using either HA assay (for H1N1) or plaque assay (for MERS-CoV). A volume of 15 mL of the inactivated viral harvest was then carefully layered with 6 mL of 20% sucrose in an ultra-centrifugation tube and centrifuged in a Sorvall MTX 150 ultracentrifuge (Thermo Scientific, CA, USA) at 28,000 rpm for 2 h at 4 °C. The pellets were further resuspended in 500 µL 1× PBS. The required amounts of viral antigen (µg) of each virus were mixed with Imject Alum adjuvant (Invitrogen, CA, USA) in a ratio of 1:1 (*v*/*v*). The final antigen/adjuvant combination was continuously mixed for 30 min under cooling conditions to effectively adsorb the antigen into the surface of the adjuvant and generate optimal vaccine formulation.

### 2.6. Immunization of Mice

Female BALB/c mice (6–8 week-old) were reared and supplied from the animal house at the National Research Centre (NRC), Egypt. Mice were divided into 5 groups (7 mice/group). Two groups of mice were intramuscularly injected with 5 µg of OMVs-H1/RBD and OMVs-Empty. Three other groups were used as controls including negative control group that was injected with sterile PBS and two positive control groups that were injected either with inactivated H1N1pdm09 or inactivated MERS-CoV. All animals received booster immunizations after 3 weeks. Serum samples were collected at 0, 2, 4, 6, and 8 weeks after prime immunization. All mice sera were separated and stored at −20 °C until used.

### 2.7. Hemagglutinin Inhibition (HAI)

Sera collected from immunized/control mice were treated with receptor-destroying enzyme (RDE) from Vibrio cholerae (Denka Seiken, Tokyo, Japan) and kept overnight at 37 °C. The RDE was then inactivated by incubation at 56 °C for 1 h. Diluted sera were incubated with four HA units of H1N1pdm and a 1.0% suspension of chicken red blood cells, incubated for 1 h at RT. HAI titer is the reciprocal value of the dilution at which no agglutination was observed. Titers <1:10 were considered as negative.

### 2.8. Plaque-Reduction Neutralization Test (PRNT)

Plaque-reduction neutralization test (PRNT) assay was performed to determine the efficacy of stimulated antibodies in sera from vaccinated/control BALB/C mice to neutralize MERS-CoV. Briefly, sera were inactivated by heating at 56 °C in a water bath for 30 min. Sera were diluted two-fold serial dilution from 1:20 to 1:160 dilution in 40 µL of DMEM/2% FBS. An equal amount of plaque forming unit in 40 µL DMEM/2% FBS was added over sera dilutions. The serum/virus dilutions were then incubated at 37 °C for 1 h in a humidified incubator with 5% CO_2_. Afterwards, 50 µL of each dilution were inoculated into individual wells of 12-well tissue culture plates with confluent Vero-E6 cell monolayers and incubated at 37 °C for 1 h. The plates were periodically undulated every 20 min to avoid cell drying. After 1 h of virus adsorption, inoculum was removed gently from the infected monolayer cells, washed with 1× PBS and covered with an overlay containing 1× MEM media, 1% agar, 1% Penicillin/Streptomycin (Pen/Strep). The plates were left to solidify and incubated at 37 °C with 5% CO_2_ upside down until the formation of viral plaques were visible (3 days). The cell monolayers were then fixed with 3.4% formaldehyde solution for 1 h at RT, stained with 1% crystal violet solution (in 20% methanol) for 30 min at RT, and washed with water to visualize the plaques. The percent (%) of inhibition is calculated as following:

% of plaque reduction = (virus control plaques count-sample plaques count)/(virus control plaques count) × 100

The PRNT_50_ is defined as the reciprocal of the antibody dilution required to reduce the number of MERS-CoV plaques in Vero-E6 cells by 50% relative to the control wells.

### 2.9. Challenge Infection of Mice

Eight weeks after prime immunization, mice were anesthetized by intra-peritoneal injection with Ketamine solution with doses adjusted to their individual body weight (2 μg/g). An infectious dose of 10^5.5^ TCID_50_ of influenza virus A/California/04/2009 (H1N1) wild type, was administered intra-nasally to all vaccinated and control groups. To ensure full separation between the groups and the absence of natural infection, an additional control group of five mice was added and not subjected to infection. Body weight was monitored daily and mice showing a weight loss of more than 30% of their initial body weight were euthanized and recorded as dead. Mice were kept under specific pathogen free (SPF) conditions at the National Research Centre facility unit, Egypt.

### 2.10. Ethics Statement and Biosafety

All animal trials were conducted in accordance with the recommendations and guidelines of the Egyptian Animal Welfare Legislation. The ethics committee of the National Research Centre, Egypt, approved the animal trial in mice (Approval code: 16-247). All experiments with infectious virus were performed according to Egyptian regulations for the propagation of influenza viruses. All experiments involving low pathogenic and highly pathogenic avian influenza A viruses were performed in biosafety level 2 and 3 (BSL2, 3) containment cabinets, respectively, approved for such use by the local authorities.

## 3. Results

### 3.1. Design and Construction of Chimeric pMP-H1/RBD

**Figure 1:**
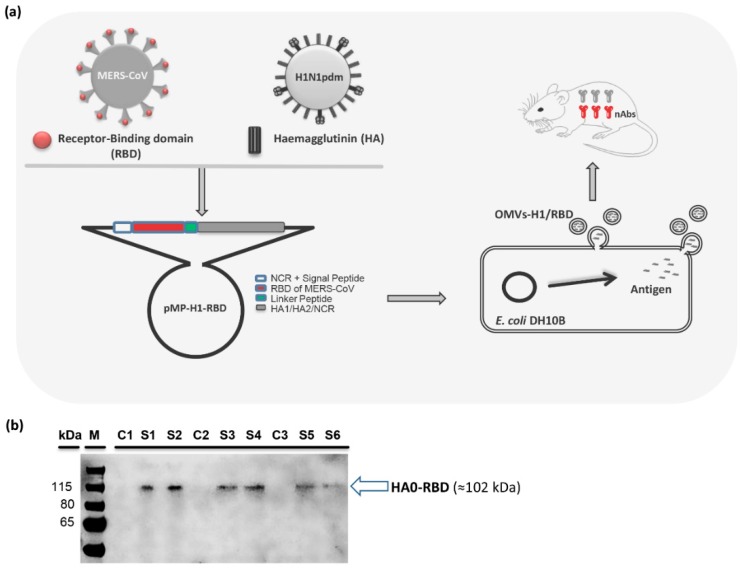
Construction of pMP-H1/RBD and characterization of the secreted OMVs-H1/RBD. (**a**) Schematic representation of the outer-membrane vesicles (OMVs)-vaccination platform to combat MERS-CoV and pandemic 2009 H1N1 (H1N1pdm09). The target sequences of MERS-CoV (RBD, 720 bp, 240 aa) and H1N1pdm09 (HA) were amplified, purified, digested and ligated to pMPccdB vector. The ligated plasmids harboring the target sequences were transformed into bacteria and the secreted OMVs containing recombinant antigens were then purified for evaluation in mice. Abbreviations: NCR: Non-coding region, SP: Signal peptide, RBD: Receptor binding domain (MERS-CoV), L: Nucleotide sequence of peptide linker (GGTAGCGCCGGTAGCGCCGGA), HA1: Hemagglutinin 1, and HA2: Hemagglutinin 2. (**b**) Immunoblotting pattern of OMVs, extracted either from various control/non-transformed OMVs (C1, C2, and C3) (empty OMVs) or pMP-H1/RBD-transformed (OMVs-H1/RBD) DH10ß (S1-S6), against antiserum of swine HA1 antibody.

As schematically represented in Figure 1a, the pMP-H1/RBD construct comprises of the 3’- Non-Coding Region (NCR) and the signal peptide (SP) of the HA gene from A/Giessen/06/2009 (H1N1pdm2009), followed by the receptor-binding domain (RBD) of the MERS-CoV spike gene (RBD: 1099-1818 nt = 367-606 amino acids (aa)), a 7-aa flexible linker peptide (LP: GSAGSAG, [22]) and the coding sequence plus the 5’-NCR of the HA gene. The pMP-H1/RBD is designed to link the RBD and HA0 fragments into a single polypeptide chain. The final fusion protein contains amino acid (aa) residues 1–240 of the RBD, aa residues 1–7 of the LP and aa residues 1-566 of the HA0.

### 3.2. Production and Characterization of OMVs with Chimeric HA/RBD Antigen (OMVs-H1/RBD)

To produce outer-membrane vesicles (OMVs), comprising an expressed MERS-CoV RBD and H1N1pdm2009 HA (OMVs-H1/RBD) hybrid protein, the *E. coli* strain DH10ß was transformed with pMP-H1/RBD plasmid. The presence of the RBD from MERS-CoV and the H1-HA from H1N1pdm2009 in the OMVs particles was examined by immunoblot analysis using 5 µg of purified OMVs-H1/RBD. The blotting pattern confirmed the presence of RBD-linked HA viral protein; corresponding to HA0-RBD (77 kDa + 25 kDa equal 102 kDa). In contrast, OMVs isolated from untransformed DH10ß (OMVs-empty) did not show any cross-reacting proteins (Figure 1b).

### 3.3. Immunization with OMVs-H1/RBD Elicits Specific IgG Titers

**Figure 2:**
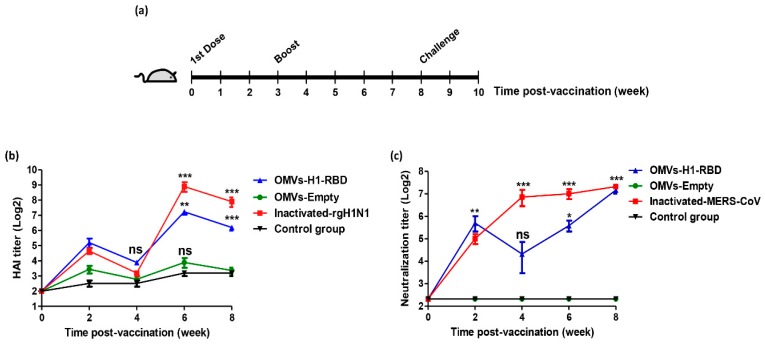
Immunostimulation of neutralizing antibodies against H1N1pdm2009 and MERS-CoV in mice. (**a**) Experimental timeline. (**b**) Hemagglutinin Inhibition (HAI) antibody titers against rgH1N1pdm09 in mice sera of vaccinated groups with OMVs-H1/RBD and inactivated H1N1pdm09 were monitored every two weeks in comparison to control PBS and OMVs-empty groups. Statistical changes marked by * *p* value < 0.05, ** *p* value < 0.01, *** *p* value < 0.001 and ns *p* value > 0.05 non-significant change. (**c**) Neutralization titer against MERS-CoV for mice sera vaccinated with inactivated MERS-CoV and OMVs-H1/RBD using plaque reduction neutralization (PRNT) assay. Statistical changes marked by * *p* value < 0.05, ** *p* value < 0.01, *** *p* value < 0.001 and ns *p* value > 0.05 non-significant change.

To assess the immunogenicity of the bivalent OMVs-H1/RBD preparations, female BALB/c mice were immunized with 5 µg/mouse of OMVs-H1/RBD and OMVs-empty in comparison with inactivated H1N1pdm2009 and inactivated MERS-CoV as a positive controls and PBS as a negative control. Mice received a booster dose three weeks after prime immunization (Figure 2a). Sera were collected every two weeks from week two to eight after prime immunization.

At two weeks post-vaccination, mice vaccinated with inactivated H1N1pdm2009 virus or OMVs-H1/RBD revealed a 2–3 × log2 increase in HAI antibody titer as compared to the control and OMVs-empty groups (Figure 2b). Four weeks after vaccination we observed a drop in the HAI titers in these mice due to the booster vaccination at week three. Interestingly, the two groups vaccinated with OMVs-H1/RBD or inactivated H1N1pdm2009 showed a significant increase in geometric mean HAI antibody titers to 145.4 (7.2 log2) and 420 (8.7 log2) at week six, and at week eight, the geometric mean HAI antibody titers decreased to 70.7 (6.1 log2) and 210 (7.7 log2), respectively. These results revealed that the vaccinated mice had developed a strong immunogenic response against the H1N1pdm2009 virus. In the negative PBS control group and in the OMVs-empty group HAI titers remained low, reflecting that all animals were indeed housed under influenza-free conditions (no natural infection).

**Figure 3:**
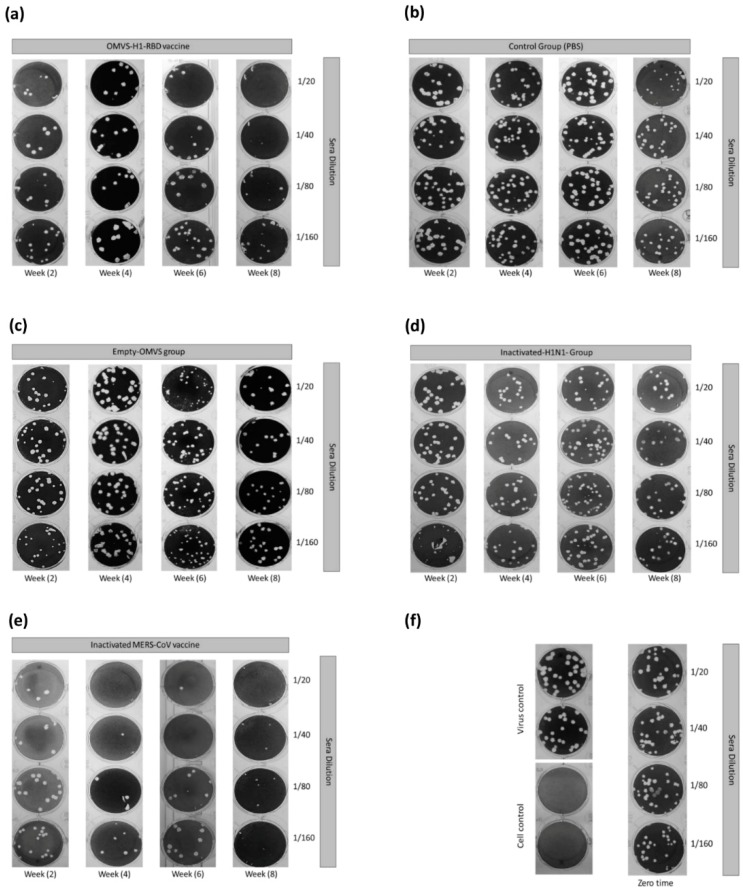
Plaque reduction neutralization (PRNT) from week 2 to week 8 in mice sera of vaccinated (**a**, **d**, and **e**) and control groups (**b** and **c**). (**f**) represents the MERS-CoV-positive control for the PRNT assay, cell negative control, and mice sera at zero time of the experiment.

In addition, the OMVs-H1/RBD vaccinated mice showed a significant increase of neutralizing antibodies against the MERS-CoV strain HKU-NRCE-270 at week 2 and reached the highest neutralizing titer 160 (7.3 log2) at week eight compared to the control group (*p* < 0.001) (Figure 2c and Figure 3a). Control (1× PBS), inactivated H1N1pdm2009 and OMVs-empty groups showed no neutralizing antibodies against MERS-CoV during the eight weeks of infection (Figure 3b–d).

On the other hand, a plaque reduction neutralization test (PRNT_50_) using sera from mice vaccinated with inactivated MERS-CoV showed complete neutralization (PRNT_50_ titer, approximately >1:160) after eight weeks of first immunization of active MERS-CoV (Figure 3e).

### 3.4. Mice Immunized with OMVs-H1/RBD Showed 100% Survival Upon Challenge with H1N1pdm09

To investigate the protection level of vaccinated mice against H1N1pdm09 infection, vaccinated- and control groups of BALB/c mice (8 weeks post-vaccination) (Figure 2a) were infected with wild type Cal-H1N1pdm2009 virus. To ensure full separation between the groups and the absence of natural infection, an additional control group of five mice was added and not subjected to infection.

**Figure 4:**
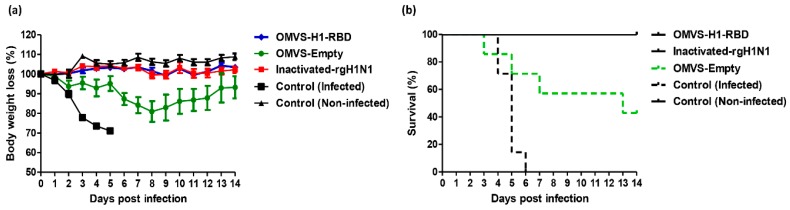
OMVs-based vaccine efficacy following challenge infection. (**a**) Body weight loss of female BALB/c mice (6–8 weeks of age) infected intra-nasally with 10^5.5^ TCID_50_ dose of Cal-H1N1pdm2009 strain. The body weight loss recorded up to 14 days p.i. and (**b**) Survival percentage at indicated time points (up to 14 days p.i.). Mice had to be euthanized when they lost ≥ 30% of their initial body weight.

Both groups, vaccinated either with inactivated H1N1pdm2009 or OMVs-H1/RBD, showed no weight losses till 14 days p.i. (post infection) in comparison to the infected PBS control group. Interestingly, these results showed that the vaccinated groups with OMVs-H1/RBD and inactivated H1N1pdm2009 virus protected all mice from Body Weight Loss (BWL) (Figure 4a) and mortality up to 14 days post challenge infection (Figure 4b).

In contrast, the control group of PBS-treated mice infected with Cal-H1N1pdm2009 100% exhibited a BWL of more than 30% from day four to six p.i. resulting in euthanasia (Figure 4a). The mortality rate in this control group was 29% four days p.i. and increased gradually to 85% at day 5 p.i., and 100% at 6 days p.i. (Figure 4b). Mortality in the OMVs-empty group reached 57% at 13 days p.i. and resulted in euthanization of 4 mice (BWL ≥ 30%) (Figure 4b).

## 4. Discussion

The continuous evolution of H1N1pdm09 in swine and human populations, and the recent emergence of MERS-CoV infections with high mortality rate in humans has raised awareness of both viruses as serious emergent global health topics [23,24].

Since vaccination is the most important strategy to combat emerging human viral infections, an effective vaccine remains a necessity, particularly for the MERS-CoV. The MERS-CoV spike (S) protein plays an essential role during virus entry through the binding of its antigenic RBD region to the DPP4 host cell receptor [25]. The RBD is recognized as a major antigenic glycoprotein fragment for inducing a potent humoral and cellular neutralizing antibody (nAb) immune responses [26,27,28,29,30,31].

Traditionally, influenza vaccines are produced by generating a natural or recombinant reassortant IAV expressing the immunogenic HA antigen [14,32]. The IAV comprises of two subunits HA1 and HA2, hosting the antigenic sites to which specific and neutralizing antibodies are elicited to combat IAVs strains during vaccination or natural infection [14,33]. However, it was reported that the specificity of the vaccine produced in cell-culture and embryonated eggs is occasionally impaired by amino acid (aa) changes, due to seed strain adaptation, with a drastic impact on vaccine effectiveness [14,15,16]. The vaccine platform presented in this study depends on plasmid-based bacterial expression of recombinant viral antigen(s). These plasmids, encoding viral antigen(s), can be easily and quickly modified to insert non-synonymous changes in the encoding region of the antigen(s). Additionally, the bacterial expression has lower mutation rates than eukaryotes [34]. This platform can be also a base for incorporating combinations of different viral antigens to address additional vaccines needed to combat seasonal H1N1, H3N2 and influenza B viruses.

OMVs had been introduced as a part of novel vaccine formulations carrying antigenic proteins eliciting protective responses in animal models from diverse microorganisms such as *N. meningitis* B, *Vibrio cholera*, *Salmonella Typhimurium*, *Pseudomonas aeruginosa*, *Gallibacterium anatis*, *Acinetobacter baumannii*, *Chlamydia trachomatis*, *Shigella spp*., and *Mycobacterium tuberculosis* [35,36,37,38,39,40,41]. LPS in the outer surface of OMVs acts as a self-adjuvant that induces humoral and cellular immunity. Therefore, OMVs vaccines may be used without extra adjuvant to increase the immunogenicity and produce antiviral innate immune responses against various influenza virus infections via activation of macrophages [42,43,44]. Despite that the exact role of LPS in the context of OMVs vaccines requires further investigations, high amounts of LPS could be a drawback due to its known endotoxicity and ability to induce excessive secretions of pro-inflammatory cytokines [45]. Therefore, several ongoing investigations aim to produce genetically detoxified and less reactogenic LPS to improve OMV safety [42,46,47]. Additionally, modified bacterial strains such as ClearColi™ BL21(DE3), which do not trigger LPS-related immune response, can be applied for OMV production [48].

Based on these observations we engineered the expression of antigenically-stable and immunogenic (OMVs)-based bivalent vaccine that elicits protective antibodies (Abs) following immunization to control infections with H1N1pdm09 and MERS-CoV. A recombinant construct comprising the HA of H1N1pdm09 fused to the RBD of the MERS-CoV S protein is expressed in an *E. coli* bacterial strain. The expressed bivalent antigens were incorporated within the released OMVs (OMVs-H1/RBD).

This novel chimeric OMVs-H1/RBD produced high levels of a neutralizing Abs titer against influenza H1N1 virus at 8 weeks post immunization. Stimulated neutralizing Abs (humoral immunity) together with LPS-induced cellular immunity could fully protect immunized mice after challenge with H1N1pdm09 without significant loss in body weight. Surprisingly, the induced non-specific cellular immunity induced by OMVs-empty could partially protect the mice. This emphasize the synergistic effect of humoral and cellular immunities secreted upon vaccination with the chimeric OMVs-H1/RBD formulation. Serum transfer experiments would be able to further elucidate the role of humoral immunity independent of cellular immunity [49].

Additionally, OMVs-H1/RBD-vaccinated mice demonstrated a significant increase in the neutralizing Abs titer against MERS-CoV (1:160) at week 8 in comparison to the control group as in Figure 2C. These findings ensured that OMV vaccination platform can provide a protection by efficient neutralization of invading H1N1pdm09 and MERS-CoV. The data presented in this study are consistent with recent reports describing the potential of OMVs as biologically active, stable and highly immunogenic vaccines to protect against IAVs. A newly developed recombinant OMVs bearing the conserved M2e protein (OMVs-M2e) from IAVs efficiently protected mice from an H1N1- and H3N2-type IAV challenge [50,51]. Our study represents an extension of these studies and suggests the generation of OMVs that incorporate combinations of different viral antigens to generate safe and efficient vaccines in animal husbandry and for humans.

## 5. Conclusions

In summary, the results show that the generated (OMVs-H1/RBD)-based vaccine presenting the antigenic stable chimeric fusion protein of H1-type HA of the pandemic influenza A virus (H1N1) strain and RBD of MERS-CoV induces specific neutralizing antibodies against H1N1pdm09 and MERS-CoV leading to protection of immunized mice against both viruses. These results demonstrate that OMVs-based vaccines presenting viral antigens have the potential to be a vaccine platform that provides simultaneous protection against two different viral infections.

## Figures and Tables

**Table 1:** Primers used in this study.

PCR Fragment	Primer Name	Primer Sequence (5’ to 3’)	Amplicon Description	Size (bp)	Ref.
Fragment (1)	F1-NcoI-PHW	CTATTACCATGGTGATGCGGTTTTGGCAGT	Part of pMP*ccd*B, and 5’-NCR/SP of HA from H1N1pdm	419	*
	R1-Bm-H1Gi-PHW	ATATCGTCTCGCTTCTGCATTTGCGGTTGCAAATG			
Fragment (2)	F2-Bm-RBD	TATTCGTCTCAGAAGCAAAACCTTCTGGCTC	RBD/peptide linker	741	*
	R2-RBD-Linker	TCCGGCGCTACCGGCGCTACCATATTCCACGCAATTGCCTA			
	R2-Bm-RBD- linker	ATATCGTCTCGTGTCTCCGGCGCTACCGGCGCTACC			
Fragment (3)	F3-Bm-HA1	TATTCGTCTCAGACACATTATGTATAGGTTA	Coding sequence of HA and 3’-NCR from H1N1pdm	1694	*
	Bm-NS-890R	ATATCGTCTCGTATTAGTAGAAACAAGGGTGTTTT			Raetz et al. 2002

Abbreviations: bp—base pair, NCR—Non-Coding Region, SP—Signal Peptide, RBD—Receptor Binding Domain, Bm—BsmBI restriction enzyme, HA1—Hemagglutinin 1, HA2—Hemagglutinin 2; * In-house primers.
